# The effect of peripheral blood lymphocyte stimulation on zeta chain expression and IL-2 production in Hodgkin's disease

**DOI:** 10.1054/bjoc.2001.1792

**Published:** 2001-05

**Authors:** I Frydecka, D Boćko, A Kosmaczewska, L Ciszak, R Morilla

**Affiliations:** Department of Haematology, Medical University, Pasteura 4, 50367 Wroclław, Poland; Laboratory of Immunopathology, Institute of Immunology and Experimental Therapy, Weigla 12, 53–114 Wroclław, Poland; Royal Marsden NHS Trust Hospital, Fulham Road, London SW3 6JJ, UK

**Keywords:** Hodgkin's disease, lymphocytes, zeta chain, stimulation

## Abstract

It has been reported that peripheral blood T cells and NK cells express reduced levels of the T-cell receptor signal-transducing zeta chain in Hodgkin's disease (HD). The zeta chain has emerged as a key subunit of the T-cell antigen receptor, which plays a central role in the signal-transducing events leading to T and NK-cell activation. We were interested in determining whether the low zeta chain expression in HD could be corrected by anti-CD3, anti-CD3-rIL-2 ex vivo stimulation. Zeta chain expression was analysed by dual immunofluorescence on permeabilized cells before and after 72 hours of culture. The IL-2 concentration in the culture supernatants was measured by ELISA. Zeta chain was significantly reduced on unstimulated CD4+, CD8+ and CD56+ cells from patients in active disease compared with normal subjects. In patients in complete remission, the values were normal except for CD8+ cells, on which zeta expression remained significantly reduced. Stimulation with anti-CD3 did not change zeta expression. Co-stimulation with rIL-2 increased but did not normalize the proportions of CD4^+^/zeta^+^, CD8^+^/zeta^+^and CD56^+^/zeta^+^cells and IL-2 production in active disease. Stimulation of cells from patients in clinical remission with anti-CD3^+^rIL-2 increased the proportion of CD8^+^zeta^+^cells and normalized IL-2 production levels. Considering the pivotal role of CD3-zeta in immune response, our data suggest that successful immunotherapy approaches in active HD should consider inclusion of other potent cytokines, as well as genetically engineered tumour vaccines. © 2001 Cancer Research Campaign www.bjcancer.com


*British Journal of Cancer* (2001) **84**(10), 1339–1343
© 2001 Cancer Research Campaign
doi: 10.1054/ bjoc.2001.1792, available online at http://www.idealibrary.com on IDEAL®

http://www.bjcancer.com

### The effect of peripheral blood lymphocyte stimulation on zeta chain expression and IL-2 production in Hodgkin's disease

**I Frydecka[1,2], D Boćko[2], A Kosmaczewska[2], L Ciszak[2] and R Morilla[3]**

[1]Department of Haematology, Medical University, Pasteura 4, 50–367 Wrocław, Poland; [2]Laboratory of Immunopathology, Institute of Immunology and Experimental Therapy, Weigla 12, 53–114 Wrocław, Poland; [3]Royal Marsden NHS Trust Hospital, Fulham Road, London SW3 6JJ, UK

**Summary** It has been reported that peripheral blood T cells and NK cells express reduced levels of the T-cell receptor signal-transducing zeta chain in Hodgkin's disease (HD). The zeta chain has emerged as a key subunit of the T-cell antigen receptor, which plays a central role in the signal-transducing events leading to T and NK-cell activation. We were interested in determining whether the low zeta chain expression in HD could be corrected by anti-CD3, anti-CD3-rIL-2 ex vivo stimulation. Zeta chain expression was analysed by dual immunofluorescence on permeabilized cells before and after 72 hours of culture. The IL-2 concentration in the culture supernatants was measured by ELISA. Zeta chain was significantly reduced on unstimulated CD4+, CD8+ and CD56+ cells from patients in active disease compared with normal subjects. In patients in complete remission, the values were normal except for CD8+ cells, on which zeta expression remained significantly reduced. Stimulation with anti-CD3 did not change zeta expression. Co-stimulation with rIL-2 increased but did not normalize the proportions of $CD4^+/zeta^+$, $CD8^+/zeta^+$ and $CD56^+/zeta^+$ cells and IL-2 production in active disease. Stimulation of cells from patients in clinical remission with anti-$CD3^+$rIL-2 increased the proportion of $CD8^+zeta^+$ cells and normalized IL-2 production levels. Considering the pivotal role of CD3-zeta in immune response, our data suggest that successful immunotherapy approaches in active HD should consider inclusion of other potent cytokines, as well as genetically engineered tumour vaccines. © 2001 Cancer Research Campaign http://www.bjcancer.com

**Keywords**: Hodgkin's disease; lymphocytes; zeta chain; stimulation

Research in tumour-induced immunosuppression has recently focused on alterations in the T-cell receptor complex (TCR/CD3) (Frydecka et al, 1998). Antigen recognition is mediated by the TCR alfa beta chains. The TCR-associated complex of CD3 transmembrane proteins provide a signal to direct the transport of the assembled TCR/CD3 complex from the Golgi apparatus to the cell surface and is involved in the transduction of activation signals following engagement of the TCR. A key role in this process is played by the zeta chain (Weissman et al, 1988; Irving and Weiss, 1991).

Natural killer (NK) cells express the zeta chain as a heterodimer linked to CD16 (Anderson et al, 1990), and the zeta chain seems to have a role in signal transduction within the NK population. This is analogous to what has been observed in T cells and may be critical for the induction of NK cell-mediated antibody-dependent cytotoxicity (Vivier et al, 1991).

The consequences of reduced zeta chain levels are likely to be functionally important because the phosphorylation of the immunoreceptor tyrosine-based activation motifs on the intracytoplasmic component of the zeta chain by protein kinases is an important early event in T-cell and NK-cell activation. Reduced levels of zeta chains result in a relative lack of tyrosine residues for phosphorylation and a reduced recruitment and phosphorylation of downstream signal-transducing molecules, such as ZAP-70, leading to a failure of T- and NK-cell activation, proliferation and cytokine production (Musci et al, 1997). Alterations in the signal transduction machinery within T cells may hamper effective activation upon recognition of tumour-associated antigens (Kiessling et al, 1999; Whiteside, 1999).

The first studies on the expression of the CD3 zeta chain in human cancer were reported by Nakagomi et al (1993) in colorectal carcinoma and Finke et al (1993) in renal cell carcinoma. After these initial studies, decreased levels of CD3 zeta and NK zeta expression were reported in several solid and haematological malignancies (reviewed in Frydecka et al, 1998; Kiessling et al, 1999; Whiteside, 1999).

Here we have extended our previous study on zeta chain expression in subpopulations of peripheral blood T cells (PBT) and NK cells in Hodgkin's disease (HD) patients (Frydecka et al, 1999). The aim was to investigate whether stimulation with anti-CD3, anti-$CD3^+$rIL-2 can overcome the previously observed impaired zeta chain expression in $CD4^+$, $CD8^+$ and $CD56^+$ cells in the active disease and $CD8^+$ cells in clinical remission and its effect on IL-2 production. According to our knowledge, no such studies have been reported so far.

### MATERIALS AND METHODS

### Patients

The study was performed in 20 patients in the active phase of HD (stages III and IV) aged 24–68 years (mean 42.0), 15 patients in clinical remission aged 25–60 years (mean 43.0) and 20 age- and sex-matched healthy subjects.

*Received 26 September 2000*
*Revised 22 February 2001*
*Accepted 28 February 2001*

*Correspondence to:* I Frydecka

The stage of the disease, defined according to the Ann Arbour classification, was assessed using standard procedures (Carbone et al, 1971). Studies were carried out with the approval of the ethical committee.

### Flow cytometric analysis

Peripheral blood mononuclear cells (PBMC) were separated by buoyant density gradient centrifugation on Gradisol L (Kutno, Poland) from freshly drawn peripheral venous blood, washed 3 times with 0.9% saline solution. All the experiments were carried out by double labelling with anti-CD4, anti-CD8, anti-CD56 monoclonal antibody (MoAb) (Becton-Dickinson, San Jose, CA) and CD3 zeta (TIA-2) MoAb directed against the cytoplasmic domain of the zeta chain (Coulter, Miami, F1). The cells were fixed and permeabilized according to the method described by Anderson et al (1980).

Briefly, the cells were incubated for 10 min at room temperature with 2 ml of a mixture of 1 ml 4% paraformaldehyde in phosphate-buffered saline (PBS) and 1 ml of a 1:10 dilution of FACS lysing solution (Becton-Dickinson) in distilled water. The cells were washed with PBS containing 0.5% Tween 20 and incubated for 20 min with AB serum and anti-zeta chain MoAb. Cells were then washed using PBS containing 0.5% Tween 20 and incubated with goat-anti-mouse FITC, washed twice with PBS/Tween 20 and resuspended for 5 min in mouse serum diluted to 1:2000. Cells were then incubated for 20 min with phycoerythrin-conjugated MoAB to CD4, CD8 and CD56, washed twice in PBS/Tween 20, resuspended in PBS, and analysed by flow cytometry using FACScalibur flow cytometer (Becton-Dickinson). Negative controls were always used, omitting the MoAb as well as incubating the cells with mouse Ig of the same isotype as MoAbs conjugated with fluoresceine or phycoerythrin.

The results were expressed as the proportions of CD4+, CD8+ and CD56+ cells co-expressing the zeta chain. At least 10 000 events per sample were analysed in double staining analysis.

### Culture conditions

PBMC were resuspended to $1 \times 10^6$ PBMC ml$^{-1}$ in RPMI 1640 supplemented with 10% fetal calf serum. Cells were stimulated with 10 ng of anti-CD3 MoAb (Ortho, Neckargemund, Germany) and 10 ng ml$^{-1}$ of anti-CD3$^+$ 500 U ml$^{-1}$ of rIL-2 (Eurocetus, Amsterdam, the Netherlands). Control cultures without stimulants were included in each experiment. The cultures were incubated at 37˚C in a humidified atmosphere containing 5% $CO_2$ for 72 h. Culture supernatants were stored at –70˚C until the time of testing.

### Measurement of IL-2 level

IL-2 concentrations were assessed in duplicate, applying a sandwich enzyme immunoassay ELISA (R&D Systems, Wiesbaden, Germany). The values were expressed as nanograms per millilitre (ng ml$^{-1}$) relative to a set of standards supplied with the kit. Basal levels of cytokine observed after culture with media and stimulants alone were subtracted from the results obtained after PBL stimulation.

### RESULTS

The proportions of lymphocyte co-expression of CD4 and the zeta chain were 61% ± 6%; 78% ± 9%; 85% ± 10% in the active disease, clinical remission and the control group, respectively (Table 1). The values for patients with active disease were statistically lower ($P$ = 0.005) compared with control group (Table 1). Stimulation with anti-CD3 did not significantly change the percentage of doubly stained cells. Co-stimulation with rIL-2 significantly increased the percentages of CD4$^+$/zeta$^+$ cells in patients with active disease ($P$ = 0.05), in clinical remission ($P$ = 0.05) and healthy subjects ($P$ = 0.05) (Table 1). However, the proportion of CD4$^+$/zeta$^+$ cells in patients with active disease remained lower compared with the healthy subjects ($P$ = 0.002) (Table 1).

The proportions of CD8$^+$/zeta$^+$ were 60% ± 8%, 69% ± 10% and 80% ± 10% in active disease, remission and healthy subjects, respectively (Table 2). The mean value of CD8$^+$/zeta$^+$ cells in active phase and clinical remission remained statistically lower ($P$ = 0.05) compared with healthy subjects (Table 2). Anti-CD3 stimulation had no effect on zeta-chain expression; co-stimulation with rIL-2 increased but did not normalize the proportion of CD8$^+$/zeta$^+$ cells in active disease, but normalized in clinical remission (Table 2).

The proportions of CD56$^+$/zeta$^+$ cells were 70% ± 8%, 81% ± 12% and 85% ± 10% in the active disease, clinical remission and

**Table 1** CD3/zeta chain expression in unstimulated, anti-CD3 MoAb stimulated and anti-CD3 MoAb + rIL-2 stimulated peripheral blood CD4$^+$ cells from patients with Hodgkin's disease in active phase of HD, complete remission and healthy controls

| | Unstimulated | Anti-CD3 MoAb stimulated | Anti-CD3 MoAb+IL-2 stimulated | *P* |
|---|---|---|---|---|
| Active disease *n* = 20 | 61% ± 6%<br>(I) | 70% ± 8%<br>(IV) | 74% ± 10%<br>(VII) | I:VII = 0.05<br>I:IV NS<br>IV:VII NS |
| Clinical remission *n* = 15 | 78% ± 9%<br>(II) | 85% ± 4%<br>(V) | 90% ± 12%<br>(VIII) | II:VIII = 0.05<br>II:V NS<br>V:VIII NS |
| Healthy controls *n* = 20 | 85% ± 10%<br>(III) | 89% ± 12%<br>(VI) | 94% ± 10%<br>(IX) | III:IX = 0.05<br>III:VI NS<br>VI:IX NS |
| *P* | I:III = 0.005<br>I:II NS,<br>II:III NS | IV:VI = 0.006<br>IV:V NS<br>V:VI NS | VII:IX = 0.002<br>VII:VIII NS,<br>VIII:IX NS | |

 © 2001 Cancer Research Campaign

**Table 2** CD3/zeta chain expression in unstimulated, anti-CD3 MoAb stimulated and anti-CD3 MoAb + rIL-2 stimulated peripheral blood CD8⁺ cells from patients with Hodgkin's disease in active phase of HD, complete remission and healthy controls

| | Unstimulated | Anti-CD3 MoAb stimulated | Anti-CD3 MoAb+IL-2 stimulated | *P* |
|---|---|---|---|---|
| Active disease *n* = 20 | 60% ± 8%<br>(I) | 65% ± 10%<br>(IV) | 72% ± 11%<br>(VII) | I:VII = 0.05<br>I:IV NS<br>IV:VII NS |
| Clinical remission *n* = 15 | 69% ± 10%<br>(II) | 74% ± 9%<br>(V) | 88% ± 13%<br>(VIII) | II:VIII = 0.02<br>II:V NS<br>V:VIII NS |
| Healthy controls *n* = 20 | 80% ± 10%<br>(III) | 87% ± 12%<br>(VI) | 89% ± 10%<br>(IX) | III:IX = 0.05<br>III:VI NS<br>VI:IX NS |
| *P* | I:II NS<br>I:III = 0.05<br>II:III = 0.05 | IV:VI = 0.04<br>V:VI = 0.05<br>IV:V NS | VII:VIII NS<br>VII:IX 0.05<br>VIII: IX NS | |

**Table 3** CD3/zeta chain expression in unstimulated, anti-CD3 MoAb stimulated and anti-CD3 MoAb + rIL-2 stimulated peripheral blood CD56⁺ cells from patients with Hodgkin's disease in active phase of HD, complete remission and healthy controls

| | Unstimulated | Anti-CD3 MoAb stimulated | Anti-CD3 MoAb+IL-2 stimulated | *P* |
|---|---|---|---|---|
| Active disease *n* = 20 | 70% ± 8%<br>(I) | 72% ± 14%<br>(IV) | 81% ± 10%<br>(VII) | I:VII = 0.02<br>I: IV NS<br>IV:VII NS |
| Clinical remission *n* = 15 | 81% ± 12%<br>(II) | 82% ± 20%<br>(V) | 88% ± 11%<br>(VIII) | II:VIII NS<br>II: V NS<br>V:VIII NS |
| Healthy controls *n* = 20 | 85% ± 10%<br>(III) | 89% ± 12%<br>(VI) | 92% ± 8%<br>(IX) | III:IX NS<br>III:VI NS<br>VI:IX NS |
| *P* | I:III = 0.05<br>I:II NS<br>II:III NS | IV:VI = 0.05<br>IV:V NS<br>V:VI = 0.05 | VII:IX = 0.05<br>VII:VIII NS<br>VIII:IX NS | |

healthy subjects, respectively. The decreased proportion of $CD56^+$/$zeta^+$ cells normalized in clinical remission (Table 3). Stimulation with anti-CD3 had no effect on zeta-chain expression in $CD56^+$ cells, but co-stimulation with rIL-2 significantly increased the mean proportion of $CD56^+$/$zeta^+$ cells in the active disease ($P$ = 0.02), though this value (81% ± 10%) was significantly lower than that obtained from healthy subjects (92% ± 8%) ($P$ = 0.05) (Table 3).

The production of IL-2 by anti-$CD3^+$rIL-2-stimulated PBMC was severely impaired in patients with active HD compared with patients in remission ($P$ = 0.05) and healthy subjects ($P$ = 0.02). In clinical remission, the mean value of the IL-2 concentration was not statistically different from that obtained in the controls (Figure 1).

### DISCUSSION

Only limited data are available concerning the effects of IL-2 on the expression of the zeta chain obtained from patients with cancer. Tartour et al (1995) reported a significant decrease in CD3 zeta chain expression in 4 out of 13 tumour-infiltrating lymphocytes (TILs) derived from renal cell carcinoma. This defect was not found after culturing TILs with IL-2. Similar results were obtained by Rabinowich et al (1996) in T lymphocytes obtained from melanoma-involved lymph nodes. In another two studies (Matsuda et al, 1995; Yoong and Adams, 1998), normalization of the level of zeta expression in colorectal cancer TIL cultures in the presence of rIL-2 has been observed. However, decreased expression of CD3 zeta was not reversed by immunotherapy with IL-2 at low or high doses in patients with melanoma (Rabinowich et al, 1996) or renal cell cancer carcinoma (Farace et al, 1994), although in some patients who achieved clinical response the initially reduced expression of zeta normalized following IL-2 therapy (Rabinowich et al, 1996). In a recent study, Guarini et al (1997) reported that transferring the IL-2 gene into the DNA of human cancer cells is capable of restoring and maintaining the expression of CD3 zeta and other molecules involved in the process of T cell-mediated tumour recognition.

In our study, anti-$CD3^+$rIL-2 stimulation of PBLs obtained from patients with active HD significantly increased the proportion of $CD4^+$/$zeta^+$, $CD8^+$/$zeta^+$ and $CD56^+$/zeta cells. However, the level of zeta chain expression remained significantly lower than that found in the control subjects. These findings suggest that, in active HD, the signal-transduction machinery is more profoundly impaired than in other malignancies, since stimulation with

*© 2001 Cancer Research Campaign*

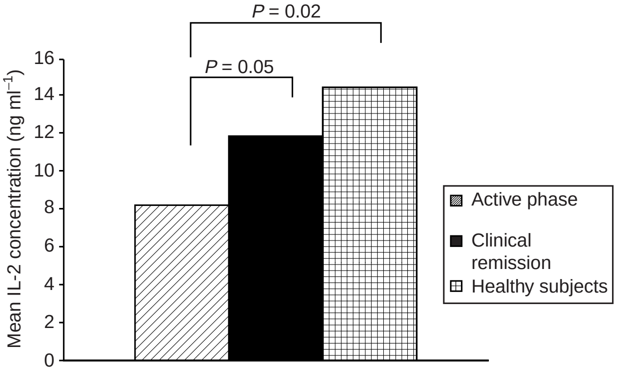

**Figure 1** The mean IL-2 concentration in supernatants obtained from PBLs stimulated with anti-CD3+IL-2 from patients with Hodgkin's disease in active disease, clinical remission and healthy subjects anti-CD3$^+$rIL-2 only partially restored the defective zeta expression (Matsuda et al, 1995; Tartour et al, 1995; Rabinowich et al, 1996; Yoong and Adams, 1998). In clinical remission, only CD8$^+$ cells showed decreased zeta chain expression, which normalized after anti-CD3$^+$rIL-2 stimulation. Our results showed that not all lymphocytes obtained from cancer patients are equally affected and that only some T immune cells could be rescued from a tumour-induced down-regulation of signalling molecules. According to current knowledge it is difficult to ascertain whether IL-2 induces the expansion of lymphocytes with a normal zeta/epsilon ratio or whether it regulates zeta expression.

However, the fact that the reversion took place rapidly (72 hours) favours the interpretation that zeta negative cells can rapidly normalize their altered CD3 complexes. As a functional correlate of the decreased zeta expression in HD, we studied the production of IL-2 by PBMC stimulated with anti-CD3+ rIL-2 in patients in the active disease and in clinical remission as compared with healthy subjects.

In our patients in active HD, stimulation with anti-CD3$^+$rIL-2 did not restore IL-2 production to normal levels, probably reflecting the persistence of a decreased proportion of CD4/zeta$^+$ and CD16$^+$/zeta$^+$ cells. In clinical remission, anti-CD3$^+$rIL-2 stimulation normalized the previously decreased zeta chain expression in CD8$^+$ cells, which probably resulted in normal IL-2 production. This finding provides indirect support for a possible clinical use of rIL-2 to prolong remission duration.

Similar results were obtained by Zea et al (1995), who found normal IL-2 production in patients with normal CD3 zeta status, but this has not been confirmed by others. Tartour et al (1995) found no correlation between IL-2 production and zeta expression in TILs and PBLs obtained from patients with renal cell carcinoma; however, the small number of patients tested does not allow drawing definite conclusions.

In summary, we have shown that rIL-2 partially restored impaired CD3 zeta expression in CD4$^+$, CD8$^+$ and CD56$^+$ cells and normalized the proportion of CD8$^+$/zeta$^+$ expression in cells from patients in clinical remission, which probably resulted in normal IL-2 production in these patients. Considering the pivotal role of CD3 zeta in the immune response, our data suggest that other immunotherapeutic approaches could be considered in HD, including the use of other cytokines and vaccinations with genetically engineered tumour vaccines.

### ACKNOWLEDGEMENT

We are grateful to Prof D Catovsky (Royal Marsden NHS Trust Hospital, London, UK) for his suggestions, helpful discussion and comments on the manuscript. This work was supported by the State Committee for Scientific Research (KBN, Poland, grant no. 1452/PO5/97)

### REFERENCES

Anderson P, Blue ML, O'Brien C and Schlossman SF (1989) Monoclonal antibodies reactive with T-cell receptor zeta chain: production and characterisation using a new method. *J Immunol* **143**: 1899–1904

Anderson P, Caligiuri M, O'Brien C, Manley T, Ritz J and Schlossman SF (1990) Fc-gamma receptor type III (CD16) is included in zeta NK receptor complex expressed by human natural killer cells. *Proc Nat Acad Sci USA* **87**: 2274–2278

Carbone OO, Kaplan HS, Mushoff K, Smithers DW and Tubiana M (1971) Report of the committee of Hodgkin's disease staging classification. *Cancer Res* **31**: 1860–1870

Farace F, Angevin E, Vanderplancke J, Escudier B and Triebel F (1994) The decreased expression of CD3 zeta chain in cancer patients is not reversed by IL-2 administration. *Int J Cancer* **59**: 752–755

Finke JH, Zea AH, Stanley J, Longo DL, Mizoguchi H, Tubbs RR, Wiltrout RH, O'Shea JJ, Kudoh S, Klein E, Bukowski RM and Ochoa AC (1993) Loss of T-cell receptor zeta and p56lck in T-cell infiltrating human renal cell carcinoma. *Cancer Res* **53**: 5613–5618

Frydecka I, Kaczmarek P, Boc̀ko D, Kosmaczewska A and Ciszak L (1998) Alterations in signal-transducing molecule CD3 zeta in patients with neoplastic diseases. *Arch Immun Ther Exp* **46**: 355–359

Frydecka I, Kaczmarek P, Boc̀ko D, Kosmaczewska A, Morilla R and Catovsky D (1999) Expression of signal-transducing zeta chain in peripheral blood lymphocytes and natural killer cells in patients with Hodgkin's disease in different phases of the disease. *Leuk Lymphoma* **35**: 545–554

Guarini A, Riera L, Cignetti A, Montacchini L, Massaia M and Foa R (1997) Transfer of the interleukin-2 gene into human cancer cells induces specific antitumour recognition and restores the expression of CD3/T receptor associated signal transduction molecules. *Blood* **1**: 212–218

Irving BA and Weiss A (1991) The cytoplasmic domain of the T cell receptor zeta chain is sufficient to couple to receptor-associated signal transduction pathway. *Cell* **64**: 891–901

Kiessling R, Wasserman K, Horiguchi S, Kono K, Sjoberg J, Pisa P and Peterson M (1999) Tumour induced immune dysfunction. *Cancer Immunol Immunother* **48**: 353–362

Matsuda M, Petersson M, Lenkei R, Taupin JL, Magnusson I, Mellstedt H, Anderson P and Kiessling R (1995) Alterations in the signal-transducing molecules of T cells and NK cells in colorectal tumour-infiltrating, gut mucosal and peripheral lymphocytes: correlation with the stage of the disease. *Int J Cancer* **61**: 765

Musci MA, Latinis KM and Koretzky GA (1997) Signalling events in T lymphocytes leading to cellular activation or programmed cell death. *Clin Immunol Immunopathol* **83**: 205–222

Nakagomi H, Petersson M, Magnusson L, Juhlin C, Matsuda M, Mellstedt H, Taupin JL, Vivier E, Anderson P and Kiessling R (1993) Decreased expression of the signal-transducing zeta chains in tumour infiltrating T-cells and NK cells of patients with colorectal carcinoma. *Cancer Res* **53**: 5610–1612

Rabinowich H, Banks M, Reichert TE, Logan TF, Kirkwood JM and Whiteside TL (1996) Expression and activity of signalling molecules in T lymphocytes obtained from patients with metastatic melanoma before, and after interleukin-2 therapy. *Clin Cancer Res* **2**: 1263–1272

Tartour E, Tartour S, Mathiot C, Thiounn N, Mosser V, Joyeux I, D'Enghien CD, Lee R, Debre B and Fridman WH (1995) Variable expression of CD3 zeta chain in tumour-infiltrating lymphocytes (TIL) derived from renal-cell carcinoma: relationship with TIL phenotype and function. *Int J Cancer* **63**: 205–212

Vivier E, Morin P, O'Brien C, Druker B, Schlossman SF and Anderson P (1991) Tyrosine phosphorylation of the FcRIII CD16: zeta complex in human natural killer cells. Induction by antibody-dependent cytotoxicity but not by natural killing. *J Immunol* **146**: 206–210

Weissman AM, Baniyash M, Hou D, Samelson LE, Burgess WH and Klausner RD (1988) Molecular cloning of the zeta chain of the T cell antigen receptor. *Science (Wash.)* **239**: 1018–1021

*© 2001 Cancer Research Campaign*

Whiteside TL (1999) Signaling defects in T lymphocytes of patients with malignancy. *Cancer Immunol Immunother* **48**: 336–352

Yoong KF and Adams DH (1998) Interleukin-2 restores CD3 zeta chain expression but fails to generate tumour-specific lytic activity in tumour-infiltrating lymphocytes derived from human colorectal hepatic metastases. *Br J Cancer* **77**: 1072–1081

Zea AH, Brendan D, Curti BD, Longo DL, Alvord GW, Strobi SL, Mizoguchi H, Creekmore SP, O'Shea JJ, Powers GC, Urba WJ and Ochoa AC (1995) Alterations in T cell receptor and signal transduction molecules in melanoma patients. *Clin Cancer Res* **1**: 1327–1335

*© 2001 Cancer Research Campaign*
